# Psychometric Comparison of the Complete and Short Form of the Persian Academic Procrastination Scale Among Iranian Students

**DOI:** 10.1002/brb3.71417

**Published:** 2026-04-14

**Authors:** Abbas Abdollahi, Fateme Nematollahi, Soolmaz Dehghanidowlatabadi

**Affiliations:** ^1^ Wellbeing Research Center UCSI University Kuala Lumpur Malaysia; ^2^ Faculty of Social Sciences and Liberal Arts UCSI University Kuala Lumpur Malaysia; ^3^ Department of Educational Psychology, Faculty of Education and Psychology Alzahra University Tehran Iran; ^4^ Department of Counseling SR.C., Islamic Azad University Tehran Iran

**Keywords:** academic procrastination, Academic Procrastination Scale, Academic Procrastination Scale and its short form (APS‐S), psychometric assessment

## Abstract

Considering the prevalence and negative effects of academic procrastination, it is important to have appropriate scales to measure it. This study examines the psychometric properties of the Academic Procrastination Scale (APS) and its short form (APS‐S) among Iranian university students. The participants included 485 students (female: 70.3%) who voluntarily participated in this study by completing online questionnaires. Besides the common and good face and content validity specification of the Persian versions of the APS and the APS‐S, construct validity and internal consistency reliability were examined for each scale separately. Although there were proper fit models, indices, and internal consistency reliability for the full‐length and short form of the APS, the five‐item APS‐S had better fit indices. Finally, the investigations revealed that the APS‐S, as recommended by the seminal version and other translations, is a better scale to assess academic procrastination among Iranian university students.

## Graphical abstract

This study compared the psychometric properties of the Persian Academic Procrastination Scale (APS) and its short form (APS‐S) among Iranian university students. Although both scales showed acceptable validity and reliability, the five‐item APS‐S demonstrated superior model fit, supporting its use as a more efficient measure of academic procrastination.

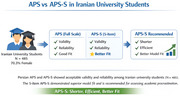

## 1 Introduction

Procrastination is derived from the Latin prefix pro‐, meaning “forward,” and crastinus, for “of tomorrow” (Merriam‐Webster 2015), and is defined as the willful postponement of a considered task in numerous settings (Ferrari and Roster 2018; Muliani et al. 2020). Academic procrastination was considered a specific type of behavioral procrastination that referred to the disposition to willingly postpone an intended course of study‐related actions (e.g., writing assigned papers, preparing for examinations, and carrying on weekly tasks) even with the negative consequences of such postponement (Abdollahi et al. 2020; Milgram et al. 1998; Steel and Klingsieck 2016). Academic procrastination is correlated with stress, self‐efficacy, achievement motivation, and academic performance (Akmal et al. 2017; Cormack et al. 2020; Itani 2019; Kim and Seo 2015), and studies have revealed that between 23% and 60% of undergraduate and graduate students frequently put off completing their academic work (Bäulke et al. 2018; Hayat et al. 2020; Onwuegbuzie 2004; Özer et al. 2009; Solomon and Rothblum 1984). However, in recent years, researchers have adopted newer approaches to the study of academic procrastination, examining the phenomenon not simply as a simple behavior but as a multidimensional construct within the framework of more complex explanatory models. In these approaches, academic procrastination has been studied in relation to psychological, educational, and especially technology‐related behaviors. For example, a study showed that multiple factors, including individual characteristics, motivational factors, and learning conditions, could simultaneously predict academic procrastination (Özbay et al. 2025). In addition, a study showed that academic procrastination acted as a mediating variable in the relationship between nomophobia (fear of not having a mobile phone) and netless phobia (anxiety caused by not having access to the Internet) (Karagöz and Özbay 2025). Such findings indicate that accurate and efficient measurement of academic procrastination has become increasingly important for testing contemporary explanatory models. Consequently, the use of valid, yet short and efficient, measurement tools, such as the full form and short form of the Academic Procrastination Scale (APS), could play an important role in advancing future research in this area. Considering the pervasiveness of procrastination among university students and its possible detrimental consequences, it is necessary to implement preventions that help students overcome procrastination (Karla Silva Soares et al. 2022; Zacks and Hen 2018). Therefore, it is necessary to have a proper scale to measure academic procrastination.

Although several measures exist to evaluate procrastination in general (Chun Chu and Choi 2005; Lay 1986; Tuckman 1991), just a few measurements were specifically created to measure academic procrastination activities. In the academic setting, two of the most popular measures for evaluating procrastination are the Procrastination Assessment Scale–Students (PASS) as the first recorded self‐report measure of procrastination developed by Solomon and Rothblum (1984), and the Tuckman Procrastination Scale (TPS; Tuckman 1991). The PASS with 12 items, which has been used in the majority of academic procrastination research (Harrington 2005; Hayat et al. 2020), has focused on procrastination on specific types of academic tasks and has been shown to have adequate reliability (*α* = 0.80). The TPS with 16 items, despite being comprised mostly of items that evaluate general procrastination in place of academic procrastination, is largely used in academic settings and is used to measure task evasion concerning academic activities. The TPS has been shown to have a proper estimate of reliability (Tuckman 1991). Despite the common use of the PASS and the TPS in academic studies, they have some limitations. McCloskey (2011) pointed out limitations such as restricting to only six areas of academic performance in the PASS and bad scale validation in the TPS and then developed the APS to resolve the stated limitations.

The APS was developed to evaluate ordinary academic procrastination, with less stress on certain kinds of academic tasks, such as postponing a project deadline (Brando‐Garrido et al. 2020). McCloskey (2011) identified six fundamental bases of academic procrastination: psychological views about capabilities, attentional diversion, social elements, time management ability, self‐initiative, and idleness. The 25‐item APS demonstrated excellent internal consistency reliability (*α* = 0.94) and admirable convergent validity in a validation study with 622 participants, with average‐to‐strong correlations with previous procrastination scales such as the PASS and the TPS. The APS consisted of one main component, covering nearly 43% of the variance. McCloskey (2011) also suggested a briefer five‐item version of the scale, picking items from the 25‐item APS scale that posed some of the most favorable psychometric properties (i.e., ITC > 0.7). Yockey (2016) examined the psychometric properties of the APS‐short form (APS‐S) in 282 university students from various ethnic backgrounds in North America, which demonstrated good internal consistency reliability (*α* = 0.87). Analysis of the APS‐S indicated its construct had one predominant component, accounting for approximately 65% of the variance.

APS has been utilized to assess academic procrastination in several studies in recent years (e.g., Akmal et al. 2017; Itani 2019; Muliani et al. 2020). Moreover, the APS was validated in Brazil (Karla Silva Soares et al. 2022) and other countries, such as Spain and India (Abdollahi et al. 2022; Brando‐Garrido et al. 2020; Chakraborty and Chechi 2019; Karla Silva Soares et al. 2022; Martín‐Antón et al. 2022), and it has been used in many research studies (Abdollahi et al. 2015; Ghayas et al. 2022; Nemtcan et al. 2022).

Therefore, the current study aimed to perform the translation of the APS from English to Persian, perform the transcultural adaptation, investigate the psychometric properties of the APS‐S and the APS‐S, and then compare their psychometric properties among Iranian university students, especially their reliability, factorial validity, and convergent validity.

## 2 Method

### 2.1 Participants

Participants were 485 Iranian university students aged 18–43 years old (*M* = 22.48, SD = 4.66), with a majority of women (70.3%), singles (80.7%), and no jobs (74.8%). Participants were studying in Iranian universities (undergraduate = 352 and postgraduate = 133); 117 (24.1%) of them were first‐year students, 98 (20.2%) of them were sophomores, 61 (12.6%) of them were juniors, and 76 (15.7%) of them were seniors.

### 2.2 Procedure of Study

Kline (2005) recommended using 5–20 subjects per item for confirmatory factor analysis (CFA). In the current study, there were 485 subjects, 25 items for the APS, and 5 for the APS‐S. So, the recommended criteria were met for both scales. The participants admitted to filling out the APS, convergent validity scales, and a demographic questionnaire. Online questionnaires were disseminated to safeguard environmental resources, and as completion of every question was required, no missing information was recorded.

The ethics committee of Alzahra University (ethical code: IR.ALU.REC.1400.015) approved the current study. The APS was translated from English to Persian using the (Brislin 1986) method. At first, the scale was interpreted from English to Persian by a linguist proficient in Persian, English, and teaching. Then, the primary Persian version was sent to a certified interpreter, who did the backward translation. Ultimately, the retranslated version was compared with the seminal English form, and their equality was confirmed. The questionnaires were prepared using a Google form, and its link was advertised on popular social media (e.g., Telegram and Instagram) to gather data. Data collection took place in November–December 2022. The online questionnaires were completed by participants in an average of 10 min.

## 3 Measures

### 3.1 Academic Procrastination Scale

The APS, as a single‐factorial scale, comprises 25 items (e.g., “I put off projects until the last minute”) and was developed by McCloskey (2011) to measure academic procrastination with a less focused view on specific kinds of assignments. Participants have to respond to the degree to which the items are compatible with them using a five‐point Likert scale (1 = disagree; 5 = agree). Greater scores in the APS indicate more academic procrastination. Based on a sample of 622 university students, McCloskey (2011) indicated an internal consistency reliability estimate of 0.93 for the APS.

### 3.2 APS‐Short Form

The APS‐S (McCloskey 2011; Yockey 2016) consisted of five APS items (2, 4, 7, 17, and 23), and respondents responded with the same Likert form. Yockey (2016) found that the APS‐S had a good internal consistency (*α* = 0.87) in a sample of 282 university students in North America.

### 3.3 Tuckman Procrastination Scale

As a single‐factorial scale, the TPS comprised 16 items and was developed by Tuckman (1991). It measured procrastination dispositions (e.g., “I manage to find an excuse for not doing something”). Participants had to respond to the degree to which the items were analogous to them, using a four‐point Likert scale (1 = *that is me for sure*; 4 = *that is not me for sure*). The upper score indicated a higher disposition to procrastinate. Tuckman (1991) stated good internal consistency for the TPS (*α* = 0.86). The Iranian study showed good reliability (*α* = 0.87) for the TPS (Panahipour et al. 2019). The internal consistency reliability of TPS was 0.94 in the current study.

### 3.4 Data Analyses

Since the APS‐S contains five items (2, 4, 7, 17, and 23) of the APS, most of the analyses were performed only once on the APS. Examining face validity, content validity, construct validity, and reliability were considered to evaluate the psychometric properties of the APS and the APS‐S Persian versions, because a significant portion of the psychometric analyses (including face validity, content validity, and preliminary data analyses) were conducted first on the full APS, and then the results for the common items were also used for the APS‐S. This approach was adopted to avoid duplicate analyses and maintain analytical consistency between the two scales.

First, the Persian version of the APS was investigated for face validity to determine the extent to which the respondents agreed that the APS truly represented the intended construct. Ten university students from different grades were asked to rate the simplicity, clarity, and relevance of APS items on a Likert scale ranging from 1 (*not important*) to 5 (*really important*). The impact score was used to evaluate face validity. For calculating the impact score, the average score for each item should be multiplied by the number of points greater than or equal to four. A value of 1.5 is acceptable as the minimum face value of each item. Otherwise, the item should be deleted (Hajizadeh and Asghari 2011).

Second, the content validity of each item was evaluated by scholars to determine how much it implied academic procrastination. To assess the content validity of the Persian version of the APS, the content validity ratio (CVR) for essentiality and the content validity index (CVI) for simplicity, clarity, and relevance were used (Cook and Beckman 2006). Ten experts were asked to examine each item concerning its essentiality on a three‐point Likert scale (1: *not essential*, 2: *useful but not essential*, and 3: *essential*) and the simplicity, clarity, and relevance of the items on a four‐point Likert scale (1: *not relevant at all to* 4: *highly relevant*). Given the number of experts who rated the essentiality of each item, the CVR was computed with the below formula, and the CVI for each issue (simplicity, clarity, and relevance) about each item was computed by dividing the number of three or four scores by the number of experts (Cook and Beckman 2006):

$$\mathrm{CVR}=\frac{N_{e}-\frac{N}{2}}{\frac{N}{2}}$$

If the value of CVR is greater than the Lawshe table value of 0.62 and the CVI value is equal to or >7, it can be concluded that the items have acceptable content validity (Polit et al. 2007).

Third, two CFAs and convergent validity were used to evaluate the construct validity of the Persian versions of the APS and the APS‐S. CFA analyses with maximum likelihood (ML) estimation were conducted to assess factor loadings and measurement fit indices. Given that data from Likert scales are ordinal in nature, the use of the ML method is considered appropriate when the sample size is relatively large, and the data distribution is approximately normal (Li 2016). In this study, given the large sample size (*n* = 482) and the results of the normality test, the use of the ML method was deemed appropriate for estimating model parameters. By acquiring any factor loading >1, negative, or <0.3, the correspondence item had to be eliminated (Gorsuch 2015). The measurement fit indices were suitable for the assessment of the model fit if CMIN/df < 5; goodness of fit index (GFI) > 0.90, root mean squared error of approximation (RMSEA) < 0.08, Tucker–Lewis index (TLI) > 0.90, and comparative fit index (CFI) > 0.90 (Byrne 2013). According to Byrne (2013), if three of the measurement fit indices met the cut‐off criteria, it would be concluded that the model had a good measurement fit. The convergent validity via correlation (Pearson) between the Persian versions of the APS, the APS‐S, and the TPS scores was calculated. A strong correlation existed if the coefficient was between ±0.70 and ±1 (Schober and Schwarte 2018). It should be noted that missing data, outliers, and normality were checked before conducting the CFA analysis. If skewness and kurtosis fall inside the ranges of [−2 to +2] and [−5 to +5], it can be inferred that the data were normally distributed. The Mahalanobis‐*D* ^2^ was used to evaluate the outliers, and the ratio of Mahalanobis‐*D* ^2^ value to the number of variables was considered as an indicator of outlier detection. If this ratio is less than the critical value of 4, the data are considered free of multivariate outliers (Tabachnik and Fidell 2013). Three observations had values greater than the threshold Mahalanobis‐*D* ^2^ value of 4 and were removed. In order to maintain comparability between the two scales, the final dataset of 482 observations was used for both APS and APS‐S analyses.

Finally, evaluating the reliability of the APS and the APS‐S using internal consistency reliability (very good: *α* ≥ 0.9 and good: 0.8 ≤ *α* < 0.9), McDonald's omega, and composite reliability (*ω* > 0.70, CR > 0.70) were conducted (Hayes and Coutts 2020; Tabachnik and Fidell 2013). These indices indicate the degree of internal consistency of the scale items. Statistical analyses were performed using SPSS version 26 and AMOS version 24. A supplementary script was used in the SPSS environment to calculate the McDonald omega coefficient.

## 4 Results

### 4.1 Face Validity

Ten university students rated the simplicity, clarity, and relevance of the Persian version of the APS items on a five‐point Likert scale. Then the impact scores for each of the 25 items were calculated. The values of face validity for simplicity, clarity, and relevance for all items were >1.5. Therefore, items remained on the scale. Given that the APS‐S items were chosen from the APS items, their face validity for the Persian version of the APS‐S items was >1.5.

### 4.2 Content Validity

The calculated CVR and CVI are shown in Table 1. The results pointed out that the values of the CVR for all items were more or equal to the value of the Lawshe table (i.e., 0.62 for 10 experts). Moreover, the CVI for all items was >0.7. Therefore, the criteria of content validity for the Persian versions of the APS and the APS‐S were acquired.

**TABLE 1:** Content validity ratio (CVR) and content validity index (CVI) of the Academic Procrastination Scale (APS) and the APS‐short form (APS‐S) items.

No.	Items’ contents (^*^: reverse‐scored items)	CVR	CVI		
		Essentiality	Simplicity	Clarity	Relevance
1	I usually allocate time to review and proofread my work^*^	1	1	1	1
2	I put off projects until the last minute	1	1	1	1
3	I have found myself waiting until the day before to start a big project	0.8	1	0.9	1
4	I know I should work on school work, but I just don't do it	1	1	1	1
5	When working on school work, I usually get distracted by other things	1	1	1	0.9
6	I waste a lot of time on unimportant things	1	1	1	1
7	I get distracted by other, more fun, things when I am supposed to work on schoolwork	1	1	1	1
8	I concentrate on school work instead of other distractions*	0.8	1	1	1
9	I can't focus on school work or projects for more than an hour until I get distracted	0.8	1	1	1
10	My attention span for schoolwork is very short	0.8	1	1	1
11	Tests are meant to be studied for just the night before	0.6	1	1	0.9
12	I feel prepared well in advance for most tests^*^	0.6	1	1	1
13	“Cramming” and last‐minute studying is the best way that I study for a big test	0.8	1	1	0.9
14	I allocate time so I don't have to “cram” at the end of the semester*	1	1	1	1
15	I only study the night before exams	0.6	1	1	1
16	If an assignment is due at midnight, I will work on it until 11:59	0.8	0.9	0.9	1
17	When given an assignment, I usually put it away and forget about it until it is almost due	1	1	1	1
18	Friends usually distract me from school work	0.6	1	1	1
19	I find myself talking to friends or family instead of working on school work	0.8	1	1	1
20	On the weekends, I make plans to do homework and projects, but I get distracted and hang out with friends	0.6	1	1	1
21	I tend to put off things for the next day	0.8	1	1	1
22	I don't spend much time studying school material until the end of the semester	0.6	1	1	1
23	I frequently find myself putting important deadlines off	1	1	1	1
24	If I don't understand something, I'll usually wait until the night before a test to figure it out	1	0.9	0.9	0.9
25	I read the textbook and look over notes before coming to class and listening to a lecture or teacher^*^	0.8	1	1	0.9

### 4.3 Construct Validity

#### 4.3.1 Preliminary Analysis

Before implementing the CFA, preliminary analyses were done. As the Google form with mandatory responses had been used, no missing data existed, and the results of normality investigation in the AMOS software depicted that the skewness (−0.80 to 0.72) and the kurtosis (−1.30 to −0.40) for the Persian version of the APS and the skewness (−0.24 to 0.30) and the kurtosis (−1.28 to −1.15) for the Persian version of the APS‐S pointed out the normally distributed data.

To define outlier status of the data, the Mahalanobis distance analysis of the AMOS software was used. The largest observed Mahalanobis‐D2 to APS number of items (25) ratio was 2.99 (2.99 < 4) and confirmed the absence of any outlier in the set of data. The same analysis was conducted for the items of APS‐S. Three of the entries in the dataset had a Mahalanobis‐*D* ^2^ to APS‐S number of items (5) ratio >4, so they were removed. Consequently, to have the possibility of both scales being compared, the APS and APS‐S analyses were conducted with 482 entries out of the primary dataset.

#### 4.3.2 Confirmatory Factor Analyses

By doing the preliminary analyses, 482 entries in the dataset were used for the implementation of the CFA with ML estimation for the Persian version of APS and APS‐S (Kline 2005) in the AMOS software. Table 2 shows the mean and the factor loading corresponding to each item of the scales achieved by CFA.

**TABLE 2:** The factorial construct of the Persian version of the Academic Procrastination Scale (APS) and APS‐short form (APS‐S).

Item	APS				APS‐S			
	Mean	SD	Factor loadings	*h* ^2^	Mean	SD	Factor loadings	*h* ^2^
1	2.77	1.24	0.51	0.26				
2	3.24	1.37	0.77	0.59	3.24	1.37	0.81	0.66
3	2.27	1.28	0.67	0.45				
4	3.21	1.41	0.84	0.70	3.21	1.41	0.85	0.73
5	3.30	1.32	0.62	0.39				
6	3.34	1.31	0.66	0.43				
7	3.25	1.33	0.73	0.54	3.25	1.33	0.71	0.50
8	3.11	1.1	0.56	0.31				
9	2.92	1.40	0.48	0.23				
10	3.14	1.39	0.78	0.61				
11	2.27	1.38	0.52	0.27				
12	3.26	1.20	0.49	0.24				
13	2.39	1.37	0.46	0.21				
14	3.19	1.31	0.72	0.52				
15	2.56	1.37	0.67	0.45				
16	3.30	1.35	0.47	0.22				
17	2.68	1.42	0.76	0.58	2.68	1.42	0.77	0.60
18	2.26	1.24	0.31	0.10				
19	2.58	1.31	0.58	0.33				
2z0	2.66	1.32	0.62	0.38				
21	3.29	1.39	0.79	0.63				
22	3.17	1.36	0.83	0.68				
23	2.75	1.37	0.80	0.64	2.75	1.37	0.79	0.63
24	2.45	1.36	0.67	0.45				
25	3.83	1.23	0.47	0.22				

*Note: h* ^2^ = communalities.

The result of the CFA for the APS demonstrated that all factor loadings included were <1 and >0.31 (Gorsuch 2015), changing from 0.31 (Item 18) to 0.84 (Item 4). The measurement fit indices supported the unidimensional construct (McCloskey 2011) with the proper values (CMIND/DF = 3.37, CFI = 0.911, TLI = 0.90, RMSEA = 0.070). Cronbach's alpha (*α*), CR, and McDonald omega (*ω*) were 0.95, 0.91, and 0.95, respectively, indicating excellent internal consistency. The mean and standard deviation for the APS were 73.21 and 21.78, respectively.

All factor loadings of the APS‐S were in the range of 0.71 (Item 7) to 0.85 (Item 4). Once again, measurement fit indices (CMIND/DF = 3.03, CFI = 0.99, TLI = 0.98, RMSEA = 0.065) supported the unidimensional construct, like Yockey (2016). The Cronbach's alpha (*α*), CR, and McDonald omega (*ω*) were 0.89, 0.81, and 0.89, respectively, indicating excellent internal consistency. The mean and standard deviation for the APS‐S were 15.14 and 5.76, respectively.

#### 4.3.3 Convergent Validity

The convergent validity, Pearson's correlations between the APS, APS‐S, and the TPS scores, were acquired. Table 3 shows significant positive correlations between the APS, the APS‐S, and the TPS.

**TABLE 3:** Correlation coefficients between the scales (*n* = 482).

Variables	APS	APS‐S	TPS
APS	1		
APS‐S	0.94^**^	1	
TPS	0.81^**^	0.8^**^	1

Abbreviations: APS, Academic Procrastination Scale; APS‐S, Academic Procrastination Scale‐short form; TPS, Tuckman Procrastination Scale.

^**^ *p* < 0.01.

## 5 Discussion

Academic procrastination has considerable effects on students’ academic performance besides being correlated to various relevant variables, such as self‐esteem, stress, and fear of failure (Abdollahi et al. 2015; Akmal et al. 2017; Itani 2019; Kim and Seo 2015). Therefore, to better understand academic procrastination and design preventions to overcome it, a psychometrically sound instrument for assessing academic procrastination is needed. The APS and the APS‐S are the latest scales that concentrate on the academic setting rather than the general aspect of procrastination. The current study aimed to translate the APS‐S into Persian and then assess the psychometric specifications of the translated scales in a sample of Iranian university students.

The psychometric properties of the APS and the APS‐S were evaluated using a dataset with 482 participants. McCloskey (2011) found evidence of psychometric specification in a CFA method to support the unifactorial construct. Moreover, the evaluation of the Persian version of the APS‐S showed the unidimensional construct that Yockey (2016) proposed. The investigations of McCloskey (2011) showed that APS had one significant component with an eigenvalue >1 that covered 43% of academic procrastination's variance. A similar result was also obtained in the Brazilian version of the APS (45%) and in the Persian version (42%; current study). All of them were below the cutoff value of 50%. The same examination on the APS‐S shows similar results. In addition to the brevity of the scale, the APS‐S showed better psychometric performance than the full APS version due to the reduction of repetitive items and greater focus on the main construct of academic procrastination. The appropriate factor loadings and higher variance coverage of the APS‐S indicate that this short version, in addition to saving response time, has a more coherent and fluent structure, and its psychometric analyses are reliable for a sample of Iranian students. In Yockey's (2016) study and the current study, the APS‐S had one significant component with an eigenvalue >1, with coverage variances of 65% and 62% for academic procrastination, respectively.

As for the Brazilian versions, the evaluation also depicted an admissible model fit with good model fit indices, showing the appropriateness of its construction in the Persian culture. Both the APS and the APS‐S exhibited good reliability results (>0.70; Hayes and Coutts 2020; Kline 2005), illustrating that the evaluated scores are steady over time.

The comparison of the specifications of the Persian version of the APS and the Persian version of the APS‐S shows more adequate factor loadings and coverage of construct variance in the APS‐S compared to the APS. The lower values of the reliability coefficient in the APS‐S with respect to the APS (e.g., CR: 0.81 vs. 0.91), as McCloskey (2011) himself has pointed out, indicate several items on the APS were highly similar, and the high‐reliability coefficient could show the presence of redundancy in the scale. So, some of the items evinced a certain amount of redundancy, so the idea of a shorter form of the APS was submitted as Yockey (2016) conducted its psychometric investigation. As the APS is rather long (25 items), the APS‐S, with only 5 items and good psychometric specifications, is a sound selection. It is obvious from the number of ASP‐S translations into other languages. Critically, some items in the APS showed redundancy, and the high reliability coefficient could be due to unnecessary repetition. By eliminating redundant items, the APS‐S provides the same information with fewer items and higher psychometric focus. These features make the APS‐S an efficient tool for researchers and psychologists in the Iranian educational environment to assess academic procrastination and allow for more accurate interpretation of the results.

Examining the convergent validity of the APS and APS‐S with the TPS measure revealed a positive correlational result, highlighting the multifaceted nature of procrastination. The results regarding the convergent validity of the APS and APS‐S, which were obtained through positive correlations with the TPS, in addition to confirming the conceptual overlap between these instruments, could also be interpreted within a broader theoretical framework of procrastination. The significant correlation between these scales indicates that academic procrastination, as a specific manifestation of behavioral procrastination, is structurally related to the general construct of procrastination, but at the same time reflects specific contextual characteristics in the academic environment. From this perspective, the observed convergent validity does not simply indicate the similarity between the measurement instruments but also indicates that academic procrastination could be used as a key construct in multivariate explanatory models. In recent studies, academic procrastination has been examined not only as a behavioral outcome but also as a mediating variable in the relationships between psychological and behavioral variables. For example, modeling studies have shown that this construct could mediate the relationships between individual factors, technology‐related behaviors, and academic outcomes (Karagöz and Özbay 2025; Özbay et al. 2025). In such frameworks, which often use structural equation modeling or mediation analyses, it is important to have valid and efficient measurement instruments. Therefore, the convergent validity evidence obtained in the present study not only confirms the validity of the construct of academic procrastination but also suggests that the APS, and especially its short version, could be appropriate measures for use in contemporary model‐based research that examines the complex relationships between academic procrastination, digital behaviors, and educational outcomes.

This consequence highlights the importance of having suitable scales in an academic setting to measure academic procrastination. It prepares the way for an in‐depth study of procrastination and its impacts in an academic setting.

In addition to confirming the psychometric properties of the APS and APS‐S, the findings of the present study could also be interpreted in the context of recent research examining more complex explanatory models of academic procrastination. In recent years, some studies have attempted to examine academic procrastination in the form of multivariate models in which individual, educational, and behavioral factors simultaneously play a role in the formation of this phenomenon. For example, modeling research conducted by Özbay et al. (2025) showed that a set of psychological and contextual factors could simultaneously predict academic procrastination in students. The results of some studies have also shown that academic procrastination played a mediating role in the relationships between technology‐related variables; for example, Karagöz and Özbay (2025) showed that academic procrastination mediated the relationship between nomophobia and netless phobia in nursing students. In such studies, which often use structural equation modeling or mediation analyses, the use of short yet valid measurement tools is of particular importance. In this context, the results of the present study, which show the favorable psychometric performance of the APS‐S, indicate that this short version could be an efficient measure for use in future model‐based studies, because it facilitates its use in multivariate studies that include multiple constructs while maintaining measurement accuracy. Furthermore, such measures could also be used in intervention research, especially in studies that aim to design and evaluate educational or psychological programs to reduce academic procrastination and improve students’ academic outcomes in digital learning environments.

This study had several limitations that may impact its interpretation. First, only internal consistency and CR were considered in the APS and the APS‐S reliability investigation. Conducting the test‐retest reliability as a stage of the psychometric procedure confirms the stability of the measures. Another area for improvement of this study was the inequality in the number of men and females that was uncontrollable concerning the form of data collection. The present study also has additional limitations such as nonrandom sampling, self‐reported data collection, and gender imbalance of participants. Consideration of these limitations is essential for designing future research and interpreting the results more accurately. Considering the mentioned limitation in designing future research may lead to a better understanding of academic procrastination.

The present study examined the psychometric properties of the Persian version of the APS scale and the APS‐S scale for assessing academic procrastination in a sample of Iranian university students. The findings indicate that the APS‐S scale has better consistency for this assessment in the Iranian students studied than the APS scale. Researchers and psychologists could use the APS‐S scale in Iranian educational settings to assess academic procrastination in Iranian students, and future research could examine the validity of this scale in other cultures.

## Author Contributions

**Fateme Nematollahi** and **Abbas Abdollahi** wrote, analyzed, and interpreted the data based on content areas, reaching a consensus on classifications. All authors read and approved the final manuscript. **Soolmaz Dehghanidowlatabadi** supervised the article and revised the manuscript.

## Funding

The authors have nothing to report.

## Ethics Statement

The ethics committee of Alzahra University examined and approved the current study.

## Consent

Informed consent was obtained from all individual participants in the study. Informed consent was obtained from all individual participants in the study.

## Conflicts of Interest

The authors declare no conflicts of interest.

## Data Availability

Data are available on figshare repository at https://doi.org/10.6084/m9.figshare.31724872. Although the syntax or code for the analyses is not included, all analyses were performed with SPSS 26 and AMOS 24 software, and the data are in an open repository, so researchers can reproduce the results.
